# Clara cell 16 kDa protein: an important marker for COVID-19 severity

**DOI:** 10.3389/fimmu.2025.1527377

**Published:** 2025-04-01

**Authors:** Tineke Kardol-Hoefnagel, Bart Luijk, Leon Reteig, Saskia Haitjema, Helen L. Leavis, Henny G. Otten

**Affiliations:** ^1^ Center for Translational Immunology, University Medical Center Utrecht, Utrecht, Netherlands; ^2^ Department of Respiratory Medicine, University Medical Center Utrecht, Utrecht, Netherlands; ^3^ Utrecht Patient Oriented Database, University Medical Center Utrecht, Utrecht, Netherlands; ^4^ Department of Rheumatology & Clinical Immunology, University Medical Center, Utrecht, Netherlands; ^5^ Central Diagnostic Laboratory, University Medical Center Utrecht, Utrecht, Netherlands

**Keywords:** COVID-19, CC16, disease severity, ICU, prognostic biomarker

## Abstract

The coronavirus disease 19 (COVID-19) is a disease caused by the severe acute respiratory syndrome coronavirus 2 (SARS-CoV-2) that invades lung epithelial cells and can lead to severe respiratory failure. In this study, we evaluated whether Clara cell 16 kDa protein (CC16), a serum marker of lung alveolar cell damage, is predictive for disease severity. Patients suspected of SARS-CoV-2 infection were included in this study. Serum levels of Clara cell 16 kDa protein (CC16), soluble Fas Ligand, cytochrome C, thymus- and activation regulated chemokine (TARC) and of oxidate stress related proteins were analyzed. Clinical patient data were extracted from the Utrecht Patient Oriented Database. COVID-19 positive patients were divided in two groups according to disease severity. The mean day difference between COVID-19 diagnosis date and sampling date was +11 days. Concentrations of TARC were lower in COVID-19 positive versus COVID-19 negative patients (unpaired t-test, *p*=0.002). In addition, CC16 serum levels were significantly elevated in sera taken from patients that were admitted at the intensive care unit (ICU) (*p*=0.0082). In a matched cohort, sera taken prior to ICU admission (-3 days) contained higher CC16 levels (paired t-test, *p*=0.0072). Multivariable analyses adjusted for known risk factors (age, gender, blood counts, lactate dehydrogenase, c-reactive protein, underlying disease) showed that CC16 levels were independently associated to COVID-19 severity (interquartile-range, odds ratio 1.53, *p*=0.0102). In conclusion, our findings highlight CC16 as a promising biomarker for early identification of severe COVID19 cases, which could improve patient management and resource allocation.

## 1 Introduction

Since December 2019, a new pandemic infectious respiratory disease emerged in China, named coronavirus disease 19 (COVID-19). The disease is caused by the severe acute respiratory syndrome coronavirus 2 (SARS-CoV-2). The main target of SARS-CoV-2 is the respiratory system, mainly invading alveolar epithelial cells. The majority of patients (80%) develop mild disease, including fever, cough, and dyspnea as the most prevalent symptoms. Patients that develop severe disease require oxygen therapy and intensive care unit (ICU) admission, with respiratory failure from acute respiratory distress syndrome and/or multi-organ dysfunction (1–3).

Several studies reported a higher age and comorbidities such as diabetes mellitus, cardiovascular disease, obesity, and chronic lung diseases (4) as a risk factor for COVID-19 disease severity. Some immunological markers, like elevated serum levels of interleukin-6 and -10, seemed to be predictive for disease severity. Furthermore, a positive correlation has been found between elevated C-reactive protein (CRP) levels and severe disease (4, 5). Lower ferritin, D-dimer, lactate dehydrogenase (LDH), and aspartate transaminase have all been associated with a lower mortality risk in patients who required oxygen therapy (6). Patients with severe and fatal disease had increased neutrophil counts, and decreased lymphocyte and platelet counts (5).

Few of these markers are directly linked to lung damage, which could be a consequence of the infiltration of SARS-CoV-2 inside the lungs. A literature search was performed to look which markers could be related to lung injury, and which of these markers had not yet been studied in the context of COVID-19 disease severity. The following markers were found to be of interest: Clara cell 16 kDa protein (CC16), soluble Fas Ligand, cytochrome c, oxidative stress products, and CCL17 or thymus- and activation-regulated chemokine (TARC).

CC16 is a secretory protein produced primarily by bronchiolar club cells, and has extensively been studied as a prognostic biomarker for epithelial cell damage. Human sino-nasal epithelial cells also express CC16 (7), and a subset of hematopoietic stem cells (8), involved in airway epithelial renewal. High CC16 serum concentrations have been found in patients with acute lung injury (pulmonary fibrosis) (9) and in critical care patients with acute respiratory distress syndrome (10). Although few studies have examined CC16 as a prognostic marker for COVID-19 severity, it may be associated with worse outcomes (11–13). Furthermore, low levels of CCL17 or TARC, that plays a role in inflammatory lung injury, have been identified as a predictive marker to distinguish mild/moderate COVID-19 disease patients from severe/critical disease patients (14).

In addition to alveolar damage, one of the hallmarks of a viral infection, including COVID-19, is the development of oxidative stress (excessive reactive oxygen species (ROS) and reactive nitrogen species (RNS) production) (15). For example, oxidative stress resulted in increased production of sFasL. Increased levels of sFasL have been found in patients with different pulmonary diseases, including interstitial lung diseases and fibrotic lung diseases, and in patients with ARDS (16). Serum sFasL protein levels were decreased in COVID-19 patients compared to healthy controls (17, 18). In addition to sFasL, cytochrome c, a 14 kDa protein, can also be released upon oxidative stress, resulting in apoptosis. Cytochrome components activity is increased in patients with ICU-ARDS patients as well as in COVID-19 patients (19).

Given the role of oxidative stress in viral infections and CC16 as a biomarker for lung damage, this study evaluated CC16, CCL17/TARC, sFasL, cytochrome C, and ROS/RNS as potential predictors of COVID-19 severity. To our knowledge, no studies have been performed investigating whether these biomarkers can be used to predict a severe disease course.

## 2 Materials and methods

### 2.1 Patients

A total of 220 patients suspected positive for SARS-CoV-2 RNA were enrolled in the study. All patients evaluated for autoimmunity whom entered the University Medical Center (UMC) Utrecht between April 2020 and June 2021 were included. If there was no hospital admission, patients were excluded from the analysis. The patient cohort is a subgroup of all COVID-19 positive patients who have been admitted to the UMC Utrecht. Blood samples were centrifuged for 5 min at 2,000 x g to separate the serum. Residual biological material was stored at -20°C until further analysis.

Patients without a proven SARS-CoV-2 RNA real-time polymerase chain reaction (RT-PCR) positive test were defined as COVID-19 negative patients. Patients with a proven PCR positive test were categorized into groups according to their ICU admission status. Patients were either transferred from the ICU of other facilities (n=6), or admitted to the ICU because of acute respiratory insufficiency (n=6) or presence of acute respiratory distress syndrome (n=2). All patients required intubation.

### 2.2 Ethical approval

The study was approved by the ethics committee for biobanks at the UMC Utrecht (TCBio; reference number: 21-547, approval date 23 September 2021).

### 2.3 Clinical data extraction

For this study data from the Utrecht Patient Oriented Database (UPOD) were used. UPOD is an infrastructure of relational databases comprising of data on patient characteristics, hospital discharge diagnoses, medical procedures, medication orders and laboratory tests for all patients treated at the University Medical Center Utrecht (UMC Utrecht) since 2004. UPOD data acquisition and management is in accordance with current regulations concerning privacy and ethics. The structure and content of UPOD have been described in more detail elsewhere (20).

### 2.4 ELISA procedures

Serum levels of the following markers were determined by ELISA according to the manufacturer’s instructions: 1) human cytochrome C (Elabscience, Texas, USA); 2) SCGB1A1/Uteroglobin (alternative name CC16) (Boster Bio, California, USA); 3) human FasL (Thermo Fisher Scietific, Massachusetts, USA); 4) CCL17/TARC (R&D systems, Minneapolis, USA). Briefly, for cytochrome C and CC16, 100µl of serum was added to the wells. After 90 minutes incubation at 37°C, the serum was removed from each well, and 100µl biotinylated detection antibody was immediately added and incubated for another 60 minutes. For FasL, precoated microwell strips were washed twice, followed by addition of 50µl of serum to the wells. Immediately, 50µl of Biotin-Conjugate was added to the wells, and incubated for 2 hours at room temperature on a MTS 2/4 digital microtiter shaker (IKA, Staufen, Germany). For CCL17/TARC, 100µl of Assay Diluent RD1W was added to the wells, together with 50µl of sample. The samples were incubated for 2 hours at room temperature. After washing, conjugate working solution was added and incubated for 30-60 minutes. Unbound conjugate was washed away, followed by incubation for 10-30 minutes with Substrate Reagent. Finally, Stop Solution was added. The optical density was measured on a SpectraMax M3 microplate reader (Molecular Devices LLC, California, USA) at a wavelength of 450nm.

### 2.5 ROS/RNS assay

Total ROS and RNS were detected in serum by an OxiSelect *in vitro* ROS/RNS assay (Cell Biolabs Inc, California, USA). A proprietary fluorogenic probe, DCFH-DiOxyO, is primed with a dequenching reagent to the highly reactive DCFH form. In the presence of ROS and RNS, the DCFH is rapidly oxidized to the highly fluorescent DCF. The assay was performed according to manufacturer’s instructions. Briefly, 50µl of serum was added together with 50µl of Catalyst. Samples were mixed and incubated for 5 minutes at room temperature, followed by addition of 100µl of DCFH solution. This suspension was incubated for 30 minutes in the dark. Fluorescence was read on a SpectraMax M3 microplate reader at a wavelength of 480 nm (excitation) and of 530 nm (emission).

### 2.6 Statistical analysis

All statistical tests were performed with GraphPad Prism Software version 9.3 and R version 4.4.0. Differences in patient characteristics between controls, non ICU admitted and ICU admitted patients were assessed by the chi-squared test for categorical variables and the one-way ANOVA test for continuous variables. Differences in serum titers were assessed via an unpaired t-test, and differences between matched patients were assessed via a paired t-test.

To identify CC16 levels as an independent biomarker for COVID-19 severity, we used an ordered logistic regression model, fit with the *rms* R package (version 6.8-0) (21), where CC16 (and covariates) were regressed on the ordinal COVID severity scale [hospitalization without oxygen supplementation (n=67), hospitalization and oxygen supplementation >5 L/min (n=69), and ICU admission (n=28)]. Odds ratios (OR) were calculated using the interquartile range. Six missing values (four for LDH; 1 for lymphocyte count, 1 for neutrophil count) were imputed with their respective medians. A *p*-value < 0.05 was considered to be statistically significant.

## 3 Results

### 3.1 Patient cohort

Serum samples were collected of 220 patients suspected of SARS-CoV-2 infection. Clinical data were not available for 27 patients (no hospitalization), resulting in 193 patients included in the final data analysis. 20 patients (10%) did not have a proven RT-PCR COVID-19 positive test result. 11 patients (5.6%) had an autoimmune disorder. Patients were divided into groups, based on their ICU admission status. Group one consists of 136 (70%) hospitalized non-ICU patients, group two of 37 (19.2%) hospitalized patients who are admitted to the ICU. 9 of these patients had a serum collection date post ICU admission, and therefore are excluded from further analysis. The mean difference in days between COVID-19 diagnosis date and sample collection date was +10 days (SD ±9) for the non-ICU group, and +12.6 days (SD ±11) for the ICU group. In the group admitted to the ICU, 14 patients had serum collected at time of ICU admission [mean difference in days between ICU admission date and sampling date was +11.9 days (SD ±9.9)], and 14 patients had serum collected before ICU admission [mean -3.3 days (SD ±3.7)]. 108 patients were transferred from another facility to the UMCU (31 from emergency room, 76 from inpatient department, 1 patient unknown); 11 patients were immediately admitted to the ICU.

Baseline characteristics were compared between groups (**Table 1**). The mean age of patients in the COVID-19 positive group (62.5y) was significantly higher, as well as the percentage of male patients (58%), compared to patients without infection (49.5y, and 45%, respectively), both characteristics known to be associated with COVID-19 disease severity. The length of the hospital stay of ICU patients was significantly increased (mean 24.9 days) compared to non ICU patients (9.5 days). Furthermore, significant more people died due to COVID-19 infection in the ICU patient group [9/28 (32%)] compared to the non ICU patient group [7/136 (5%)].

**Table 1:** Patient demographic and clinical characteristics.

Characteristics		Controls (n=20)	COVID-19+ patients		*p*-value
			non IC (136)	IC (n=28)	
Age, y, mean ± SD		49.5 ± 17.6	62.3 ± 13.3	62.8 ± 13.6	0.0006^a^
Sex, male, n (%)		9 (45)	77 (57)	18 (64)	0.4132^b^
Comorbidities, n (%)					
	Hypertension	2 (10)	47 (34.6)	11 (39.3)	0.0654^b^
	Diabetes mellitus type II	5 (25)	29 (21.3)	7 (25.0)	0.8707^b^
	Chronic pulmonary disease^c^	4 (20)	11 (8.0)	4 (14.3)	0.0625^b^
	Chronic cardiac disease	N/A	30 (22.1)	5 (17.9)	0.6212^b^
	Peripheral vascular disease	N/A	5 (3.7)	0 (-)	0.3028^b^
	Obesity	N/A	40 (29.4)	9 (32.1)	0.7737^b^
Therapies, n (%)					
	Tocilizumab	0	19 (14.0)	6 (21.4)	0.0989^b^
	Antiviral agent^d^	N/A	18 (13.2)	12 (42.9)	0.0002^b^
Blood tests					
	Lymphocytes, count (x 10^9^)/L, mean [min-max]	1.27 [0.21-2.85]	1.13 [0.15-3.40]	0.77 [0.12-2.58]	0.0185^a^
	Neutrophils, count (x 10^9^)/L, mean [min-max]	7.77 [1.98-13.32]	6.72 [0.13-18.6]	6.68 [1.63-20.37]	0.4572^a^
	Platelet, count (x 10^9^)/L, mean [min-max]	307.5 [93.3-669.4]	277.0 [6.24-597.1]	248.7 [4.75-555.0]	0.2693^a^
	Creatinin, µmol/L, mean [min-max]	73.7 [34.0-201.0]	95.9 [37.0-1005.0]	95.0 [36.0-433.0]	0.6482^a^
	C-reactive protein, mg/L, mean [min-max]	79.2 [0.5-276.0]	70.7 [0.5-299.0]	106.0 [0.5-351.0]	0.0445^a^
	Ferritin, µg/L, mean [min-max]	386.8 [26.0-1361.0]	980.1 [34.0-12459.0]	1313.0 [69.0-4073.0]	0.0996^a^
	Lactate dehydrogenase, U/L, mean [min-max]	260.4 [106.0-532.0]	328.0 [76.0-1148.0]	433.9 [145.0-1126.0]	0.0024^a^
	Hemoglobulin, mmol/L, mean [min-max]	12.3 [5.5-16.7]	13.0 [5.6-17.8]	12.8 [8.1-16.3]	0.4019^a^
Hospital stay, days, mean ± SD		14.7 ± 16.0	9.5 ± 6.9	24.9 ± 12.7	<.0001^a^
Death, n (%)		1 (5)	7 (5.1)	9 (32.1)	<.0001^b^

a One-way ANOVA for continuous variables.

b Chi-square test for categorical variables.

c Chronic pulmonary diseases including chronic obstructive lung disease (COPD), fibrosis, and cystic fibrosis (CF).

d including lopinavir/ritonavir, remdisivir, (hydroxy)chloroquine, oseltamivir, and acyclovir.

COVID-19, coronavirus disease 19, ICU, intensive care unit, SD, standard deviation.

In the ICU admitted patient group, significantly more people receive an antiviral therapy (43%, and 13% in the non ICU admitted group). Lymphocyte cell counts were significantly decreased in the patients admitted to ICU (0.77 x 10^9^/L) compared to the other COVID-19 positive patients (1.13 x 10^9^/L. Furthermore, CRP, and LDH levels were significantly increased in ICU patients (106 mg/L, and 434 U/L, respectively) compared to patients without ICU admission (70.7 mg/L, and 328 U/L, respectively).

### 3.2 ROS/RNS, Cytochrome C, and sFasL serum levels were neither detectable nor correlated to disease severity

Serum concentrations of ROS/RNS, cytochrome C and sFasL were analyzed in patient samples collected around the time of COVID-19 diagnosis. Levels of ROS/RNS could be detected in 140/220 patients. For 22 patients, clinical data were not available, resulting in detectable levels in 61% of patients (118/193) with clinical data. Most patients did have levels within the normal range (1-5µM). We observed no correlation with disease severity (unpaired t-test). Mean ROS/RNS levels in non-ICU patients was 1.89µM, and in ICU patients 2.04µM. COVID-19 negative patients had comparable levels (mean 1.52µM) (**Supplementary Figure 1A**).

Cytochrome C levels were below the detection limit in 198/220 patients. In 15 patients with clinical data cytochrome C levels were detectable, and no clear correlation to disease severity could be observed (14 patients not admitted, and 1 patient admitted to the ICU). The COVID-19 negative patients had undetectably low cytochrome C levels (**Supplementary Figure 1B**).

For sFasL serum levels, we also hardly obtained measurable levels. 13/220 patients had levels above the detection limit of the assay, of which 2 patients had no clinical data, resulting in sFasL serum concentrations >0.10ng/mL in 8 non-ICU patients, and 3 ICU patients (**Supplementary Figure 1C**).

### 3.3 CCL17/TARC and CC16 serum levels correlated to disease severity

**Figure 1:**
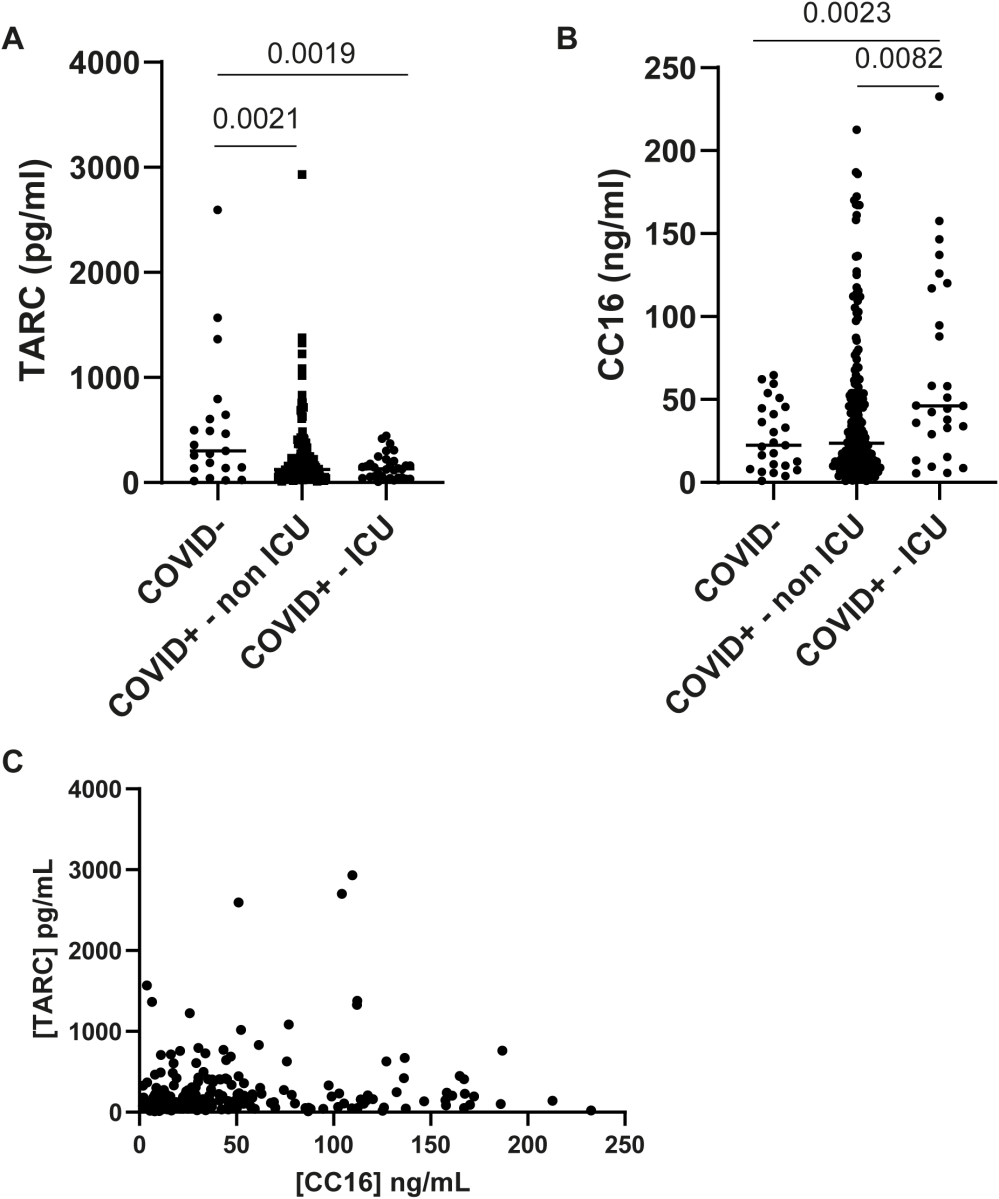
Serum concentrations in coronavirus disease 19 (COVID-19) negative patients, and in COVID-19 positive patients without or with intensive care unit (ICU) admission during hospitalization. COVID-19 positivity was proven by a real-time polymerase chain reaction. Patients were categorized according to disease severity. Non ICU was defined as no admission to the ICU at any time during their hospital stay; ICU as ICU admission at any time (early, middle or late during COVID-19 disease activity). Samples were collected prior or around the time of ICU admission. Thymus- and activation-regulated chemokine (TARC/CCL17) concentrations were measured by ELISA, and values are displayed in pg/mL (median) **(A)**. Clara cell 16 kDa protein (CC16) concentrations were also measured by ELISA, and values are displayed in ng/mL (median) **(B)**. Differences in serum concentrations between groups were analyzed with an unpaired t-test (two-tailed). Correlation between serum concentrations of CC16 and TARC **(C)** was assessed with a Pearson R test (r^2^ = 0.002, p=0.5085).

TARC/CCL17 concentrations and CC16 protein levels were measured in serum samples of 20 patients without and in 173 patients with a proven COVID-19 RT-PCR positive test. Most serum samples were collected after a proven infection (mean 9.2 days (range -3 till +34 days) after the diagnosis date). We observed lower TARC/CCL17 concentrations in COVID-19 positive patients (median serum levels are 124.4 pg/mL in the non ICU admitted patient group, and 131.7 pg/mL in the ICU admitted patient group) compared to COVID-19 negative patients (median 301.3 pg/mL) (**Figure 1A**, p = 0.002).

CC16 serum levels were significantly (*p* = 0.0023) elevated in the patients that are admitted to ICU compared to COVID-19 negative patients (median 46.15 ng/mL, and 22.47 ng/mL, respectively), and also compared to patients without ICU admission (*p* = 0.0082, 23.53 ng/mL) (**Figure 1B**).

CC16 and TARC/CCL17 levels are not correlated to each other; patients with high CC16 levels did not have low TARC/CCL17 levels or vice versa (**Figure 1C**).

### 3.4 CC16 is an independent biomarker for COVID-19 severity

**Figure 2:**
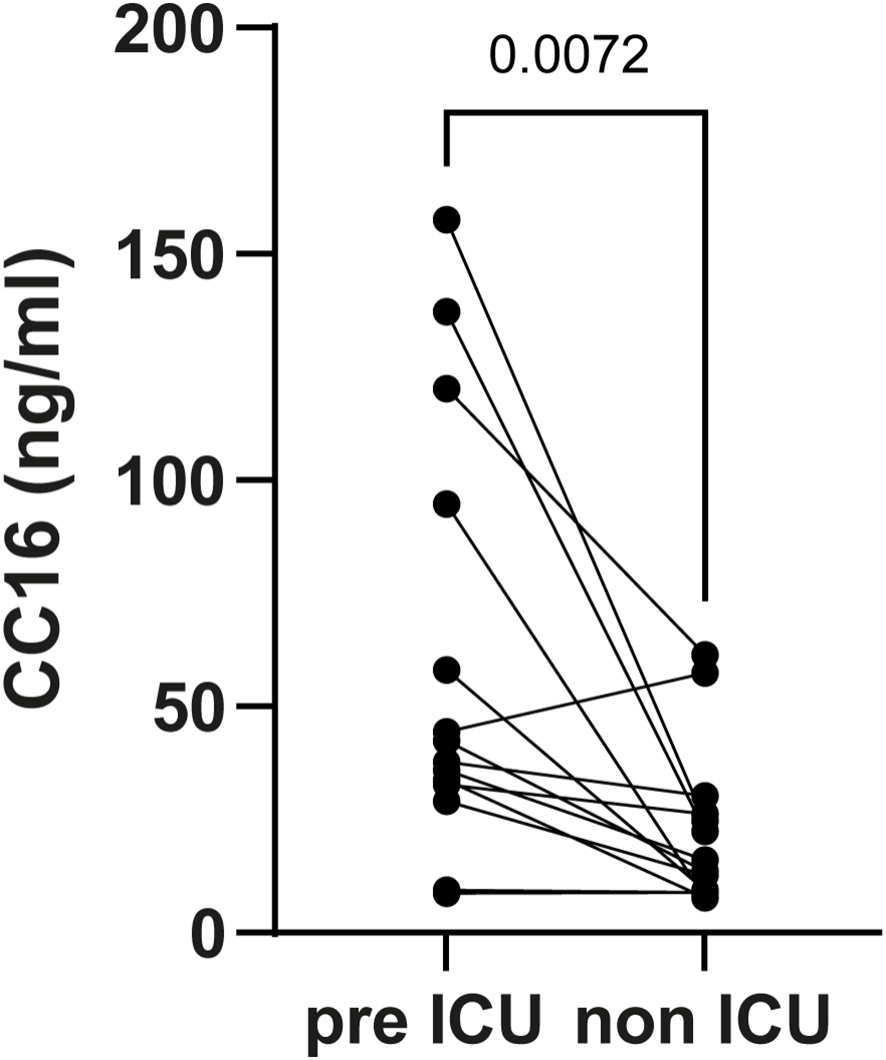
Serum Clara cell 16 kDa protein (CC16) concentrations in coronavirus disease 19 (COVID-19) positive patients collected prior to the intensive care unit (ICU) admission and in matched controls. Patients were matched for age, gender, comorbidities (hypertension, chronic pulmonary disease, diabetes mellitus type II, and chronic cardiac disease), and time between COVID-19 diagnosis and sample collection. CC16 levels were measured by ELISA, and values are displayed in ng/mL. Differences between groups were analyzed with a paired t-test (two-tailed).

To investigate whether CC16 levels can predict severe disease outcome, we compare concentrations in patients prior to ICU admission to patients without ICU admission. Patients were matched for age, gender, comorbidities (hypertension, chronic pulmonary disease, diabetes mellitus type II, chronic cardiac disease), and time from COVID-19 diagnosis. Serum CC16 concentrations were significantly higher (*p* = 0.0072) in patient samples collected on average 3.3 days before ICU admission compared to non ICU admitted patients (median [CC16] 40.09 and 14.78 ng/mL, respectively) (**Figure 2**). Furthermore, CC16 also seemed to be able to predict the outcome of patients admitted to the ICU six weeks after hospital discharge. The lowest CC16 levels were found in patients discharged home (median 32.74 ng/mL), the median levels in patients hospitalized in medium care or in a rehabilitation unit (46.30 ng/mL), and the highest levels in patients who died after infection (67.11 ng/mL) (**Supplementary Figure 2A**). CC16 levels remain high after ICU discharge (median levels 43.3, **Supplementary Figure 2B**).

In a univariable ordinal regression model, CC16 levels were significantly associated with COVID-19 severity (**Table 2**, OR 1.44, 95% CI 1.06-1.96, interquartile range 14.6-58.1 ng/mL, *p* = 0.0187). The multivariable analysis, adjusted for age, gender, levels of CRP, levels of LDH, blood lymphocyte count, blood neutrophil count, blood platelet count, presence of underlying diabetes mellitus type II, chronic pulmonary disease, and hypertension, showed that serum CC16 levels are also an independent biomarker for COVID-19 severity, as it remained significant, with a slightly larger effect size (**Table 2**, OR 1.53, 95% CI 1.11-2.11, *p* = 0.0102). All parameters were not correlated to CC16, and besides CC16, only LDH levels were significantly associated with COVID-19 severity (**Supplementary Table 1**).

**Table 2:** Uni- and multivariable analyses of the effect of CC16 serum levels on COVID-19 severity.

	Odds ratio	95% CI	*p*-value
Univariate	1.44	1.06-1.96	0.0187
Multivariable	1.53	1.11-2.11	0.0102

In this multivariable analysis we evaluated the effect of the presence of serum CC16 levels on COVID-19 severity. We adjusted for differences in the following covariates: age, sex, levels of c-reactive protein, levels of lactate dehydrogenase, blood lymphocyte count, blood neutrophil count, blood platelet count, presence of underlying diabetes mellitus type II, underlying chronic pulmonary disease including chronic obstructive lung disease, fibrosis and cystic fibrosis, and underlying hypertension. CC16, club cell secretory protein; COVID-19, coronavirus disease 19; CI, confidence interval.

## 4 Discussion

In this study, it has been shown for the first time that serum levels of CC16, a biomarker for epithelial cell damage, can be used to rule out a severe COVID-19 illness, e.g. ICU admission. In addition, CC16 levels seemed to be independently associated with COVID-19 severity. Furthermore, CCL17/TARC serum levels were lower in COVID-19 positive patients versus negative patients. Cytochrome C, sFasL or ROS/RNS serum levels seemed not to be correlated to disease severity. We found lower lymphocyte cell and platelet counts in ICU admitted patients, in line with literature (5). Increased levels of ferritin, CRP and LDH were observed in the patient group who were admitted to the ICU, compared to the non ICU admitted patient group. These findings are in line with previous studies that described the association of these biomarkers with disease severity (4–6).

CC16 is a biomarker for epithelial cell damage in the lungs. Patients with inflammation in the bronchial airways tend to have lower CC16 levels, whereas higher levels were found in patients with inflammation affecting the alveoli (13). In relation to SARS-CoV-2 infection, Tiezzi et al. (12) demonstrated that CC16 kinetics with higher serum levels on day 5 compared to day 1 upon hospitalization may have a predictive value for disease outcome. Rohmann et al. showed a positive correlation of CC16 serum levels to disease duration and activity in already severely ill COVID-19 positive as well as in sepsis patients (11). They also found a negative correlation with platelet count. We did not find such a correlation (**Supplementary Figure 3**), possibly due to the larger sample size of our cohort. Another very small cohort consisting of 7 healthy controls and 9 severe COVID-19 patients showed decreased serum CC16 levels in COVID-19. They also found reduced number of distal bronchiolar club cells, which could explain the reduced CC16 serum levels (22). In critically ill patients, SARS-CoV-2 has infected the lower respiratory tract and may cause alveolar cell damage (23). Patients with inflammatory cell damage involving the alveolar-capillary barrier, such as fibrosis or ARDS, have higher serum levels of CC16. Our results with concomitant higher levels of CC16 – even prior to the ICU admission – in more severe patients seemed to reflect alveolar involvement in COVID-19 disease progression. Our study highlighted the role of CC16 as a promising biomarker to identify severe COVID-19 cases at an early stage.

COVID-19 infection has three distinct phases: phase one is the initial viral replication phase, phase two the inflammatory lung injury phase, and phase three the post-acute sequelae phase (24). In phase one, monocytes and macrophages will migrate to the site of inflammation (lungs), and one of the triggers for monocyte/macrophage migration is CCL17 or TARC (25). Animal studies showed that mice infected with either a rhinovirus or with acute respiratory syncytial virus have higher levels of CCL17/TARC (26, 27). For SARS-CoV-2 virus infection, the opposite has been shown in a relatively small cohort. Low CCL17/TARC levels were a predictive marker to distinguish mild/moderate COVID-19 disease patients from severe/critical disease patients (14).

In addition, CCL17/TARC is a biomarker of progression in several diseases, including atopic dermatitis and asthma, attracting Th2 cells to the site of inflammation (28, 29). During viral infection and replication, a displaced Th2 response can result in Th2 cell infiltrates (30). Moreover, SARS-CoV-2 infected non-human primates with the most severe disease outcomes were shown to have higher CCL17/TARC levels, which were strongly associated with viral replication (25). However, in the current cohort, we found decreased CCL17/TARC serum levels in SARS-CoV-2 positive patients. Others found no difference in plasma CCL17/TARC levels between SARS-CoV-2 positive and negative patients, but higher plasma levels in the SARS-CoV-2 positive patients were correlated to a worser outcome (31). CCL17/TARC does not only recruit Th2 cells, but can also induce regulatory T cells. It has been hypothesized that dysfunction of regulatory T cells in the lungs could induce lung cell damage, and may contribute to the development of severe illness in SARS-CoV-2 positive patients (14, 32). More research is needed to understand the role of CCL17/TARC in COVID-19 disease development.

Viral infection could be accompanied by excessive ROS and RNS production. In turn, oxidative stress could result in increased production of soluble sFasL. In COVID-19, sFas/sFasL interactions could induce hyperinflammation, recruit immune cells, and could contribute to maintenance of neutrophil activation. sFasL itself inhibited the interaction between Fas and FasL and thereby blocked apoptosis (17, 33). In our study, we hardly detected sFasL concentrations in patient serum samples. Viral infected cells could evade apoptosis induced by cytotoxic T cells by upregulating FasL (34). On the other hand, increased apoptosis of CD4 and CD8 T cells from COVID-19 patients is correlated to increased FasL expression on T cells (35). Whether this observation is accompanied with lower Fas expression on cells is not known, nor whether it is associated with cellular apoptosis, which is a limitation of the study.

In addition to sFasL serum levels, we could detect cytochrome C levels in only a few COVID-19 patients. Literature has shown that the activity of cytochrome components is increased in COVID-19. We did not measure sFasL nor cytochrome C levels consecutively over time. It may be that the sampling time, i.e. for most patients a few days after the proven COVID-19 diagnosis date, is the reason that sFasL or cytochrome C serum concentrations were below the detection limit of the assay.

Serum CC16 levels in COVID-19+ patients were measured at a single time point, around the moment patients were hospitalized for COVID-19 infection. It could be that some patients in the non ICU admitted group may actually belong to the severe group (requiring ICU admission), but weren’t admitted due to space constraints or discussed policy that no ICU admission is desired. Furthermore, the patient cohort consisted of a specific group of patients (suspicion of autoimmunity), which could limit its generalizability, as there is some evidence that patients with pre-existing autoimmunity were more likely to have a severe disease course (36). Despite those limitations, our data show that CC16 positively correlates with disease severity, and that CC16 – besides LDH – was a significant predictor of the entire COVID-19 severity scale. In addition to CC16, other lung injury markers such as surfactant protein D, or vascular permeability markers, are known to be increased in patients with COVID-19 (37, 38). We did not include these markers in our analyses, which could potentially confound the data. Future studies should validate these findings in larger, multicenter cohorts and assess the impact of demographic and comorbid conditions on CC16 levels.

In conclusion, our study showed for the first time using a regression model, the potential of serum CC16 as a biomarker to distinguish ICU admitted from non ICU admitted patients in COVID-19 disease.

## Data Availability

The raw data supporting the conclusions of this article will be made available by the authors, without undue reservation.
