# Supplementary material for: Clara cell 16 kDa protein: an important marker for COVID-19 severity
Source: Front Immunol. 2025 Apr 1;16:1527377. doi: 10.3389/fimmu.2025.1527377 (PMC11996771; doi:10.3389/fimmu.2025.1527377)
Supplement: Supplementary file 1 [file DataSheet1.docx]

**Supplementary Material**

Manuscript Clara Cell 16 kDa Protein (CC16): An important marker for COVID-19 severity

| **Supplementary Table 1** | **Multivariable analysis on COVID-19 severity** | | |
| --- | --- | --- | --- |
|  | **of covariates included in the model** | | |
|  |  |  |  |
| **Parameter** | **Odds Ratio** | **95% CI** | ***p*-value** |
| LDH | 2.15 | 1.4-3.3 | 0.0003 |
| hypertension | 1.21 | 0.6-2.3 | 0.5666 |
| neutrophil count | 1.19 | 0.7-1.9 | 0.4674 |
| diabetes mellitus type II | 1.04 | 0.5-2.1 | 0.9210 |
| CRP | 0.99 | 0.6-1.6 | 0.9671 |
| age | 0.91 | 0.6-1.4 | 0.6614 |
| chronic pulmonary disease^1^ | 0.75 | 0.2-2.8 | 0.6683 |
| lymphocyte count | 0.71 | 0.5-1.1 | 0.1020 |
| platelet count | 0.69 | 0.4-1.1 | 0.1295 |
| sex | 0.67 | 0.4-1.2 | 0.2034 |
|  |  |  |  |
| ^1^ including chronic obstructive lung disease, fibrosis and cystic fibrosis | | | |
| COVID-19, coronavirus disease 19; CI, confidence interval; LDH, lactate | | | |
| dehydrogenase; CRP, c-reactive protein | |  |  |

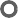

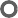

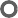

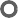

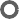

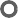

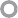

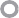

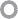

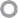

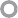

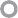

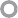

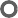

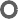

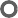

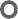

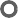

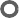

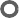

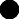

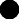

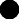

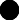

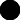

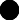

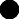

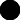

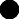


**Supplementary Figure 2**

**A**

**B**

200

0.0177

**200**

0.0638

150

**150**

100

**100**

50

**50**

median: 43.31

**0**

0

**post IC**

**CC16 (ng/ml)**

**CC16 (ng/ml)**

discharged home

hospitalization

death

**Legends**

**Figure S1.** Serum concentrations in coronavirus disease 19 (COVID-19) negative patients, and in COVID-19 positive patients with and without admission at the intensive care unit (ICU) during hospitalization. COVID-19 positivity was proven by a real-time polymerase chain reaction. Patients were categorized according to disease severity. Non ICU was defined as no admission to the ICU at any time during their hospital stay; ICU as ICU admission at any time (early, middle or late during COVID-19 disease activity). Samples were collected prior or around the time of ICU admission. Reactive oxygen species (ROS) / nitrogen species (RNS) levels were measured by an OxiSelect in vitro assay, and levels were displayed in µM (A). Cytochrome C (levels displayed in pg/mL, B) and sFasL (levels displayed in ng/mL, C) concentrations were measured by ELISA. In COVID-19 negative patients, levels for latter both biomarkers were below the detection limit of the assay.

**Figure S2.** Serum CC16 levels in ICU admitted patients and outcome after hospital discharge. Clara cell 16 kDa protein (CC16) concentrations were measured by ELISA, and values are displayed in ng/mL (median). Patients were categorized based on their status six weeks after discharge: discharged home; admitted to a hospital in medium care or in a rehabilitation unit; or death. Differences in serum concentrations between groups were analyzed with an unpaired t-test (two-tailed) (A). CC16 levels in patients post ICU admission were displayed in B.

**Figure S3.** Correlation between Clara cell 16 kDa protein (CC16) serum levels and platelet cell count. Serum CC16 concentrations of all patients were displayed in ng/mL, and correlated to platelet cell count (*10^9^/L).
